# Special series on trending themes in Neurobiology in *Biological Research*

**DOI:** 10.1186/s40659-016-0077-4

**Published:** 2016-03-17

**Authors:** Nibaldo C. Inestrosa, Manuel J. Santos

**Affiliations:** Departamento de Biología Celular y Molecular, Facultad de Ciencias Biológicas, Pontificia Universidad Católica de Chile, Santiago, Chile

A special series of timely and important articles in modern neurobiology, were put together thanks to the effort of well know Chilean neurobiologists, examining different selected themes from philosophy to molecular biology reflecting the new flavour of this particular branch from this particular field.

First, the psychiatrist Gustavo Figueroa presented an assay-review on the rather new subject “Neuroethics”, an article where he concludes that “neuroscience and its technology had progressed with such vigor that encompass, drive, configure and determine decisively the different areas of human activity” [1]. Then, Professor Iturriaga and colleagues review the function of the Carotid body (CB) chemoreceptor, and emphasize that a growing body of evidence implicates the CB in several sympathetic-mediated human diseases, including survival in heart failure, development of insulin resistance and hypertension in rat fed with a high fat diet and obstructive sleep apnea [2].

Professor Palacios and colleagues review some natural models of neurodegenerative diseases, and take advantage of bioinformatics tools, focusing especially on genetic analysis of four proteins involved in Alzheimer disease (AD) in order to explain their relationships with variants associated with the occurrence of the disease in humans [3]. Of particular interest in the natural model of AD, the caviomorph rodent *Octodon degus*, that Professor Bozinovic and colleagues develop from the cognitive and environmental point of view. In fact, they propose neuro-ecological approaches to examine how key elements of the environment may affect neural and cognitive mechanisms associated with learning, memory processes and brain structures involved in social behaviour [4]. On this context, Professor Aboitiz and colleagues describe an interesting relationship between schizophrenia and Reelin, an extracellular matrix protein in developmental connectivity and adult synaptic plasticity, and they proposed “a unifying hypothesis, that links prenatal stress and prefrontal cortex function through epigenetic alterations of the reelin gene” [5]. Finally, in a more molecular approach Professor Inestrosa and Codocedo describe that Wnt signaling, particularly Wnt-5a a key regulator of postsynaptic structures, modulates the levels of the microRNA (miR-101b), which in turn, controls the expression of cyclooxygenase (COX2) an enzyme involved in injury, inflammation and neuronal plasticity in hippocampal neurons [6].

We think this group of manuscripts forms a special series of themes that goes from neuroethics, neuronal physiology, neurogenetics, cognitive neuroscience and molecular biology, can attract the attention of the world neuroscientists towards the Chilean neurobiology community.

**Nibaldo C. Inestrosa; Guest Editor.**

**Manuel J. Santos; Editor in Chief.**
